# Multi-functional cytotoxic T cell expansion correlates with overall survival after administration of autologous dendritic cell immunotherapy in renal cell cancer patients

**DOI:** 10.1186/2051-1426-1-S1-P207

**Published:** 2013-11-07

**Authors:** M DeBenedette, I Jurisica, A Gamble, W Lewis, E Wansley, I Tcherepanova, C Nicolette

**Affiliations:** 1 Research and Development, Argos Therapeutics, Durham, NC, USA; 2 Medical Biophysics and Computer Science, University of Toronto, Toronto, ON, Canada; 3 Princess Margaret Cancer Centre, University Health Network, Toronto, ON, Canada

## Background

AGS-003 is an autologous dendritic cell immunotherapy consisting of matured DC co-electroporated with amplified autologous tumor RNA and synthetic CD40L RNA. A Phase 2 trial evaluated AGS-003-induced T cell immunity in RCC patients in combination with sunitinib. A comprehensive immune analysis using an unbiased bioinformatics approach to analyze multi-color flow cytometry data on longitudinal blood draws identified statistically significant correlates between T cell responses and overall survival.

## Results

Immune monitoring using multi-color flow cytometry was performed on subjects receiving at least five doses of AGS-003 (n=14). The large data sets where analyzed using a novel bioinformatics methodology consisting of an adaptation of binary tree-structured vector quantization (BTSVQ). BTSVQ implements two-way unsupervised clustering to partition cytotoxic T cell (CTL) subsets into related groups without consideration of clinical outcomes. This approach identified an AGS-003-induced central/memory CTL signature defined by the co-expression of CD28, CD27, and CCR7 receptors in the absence of CD45RA expression. Furthermore, increases in absolute numbers of antigen-reactive CD28+CCR7+CD27+CD45RA- CTL after five doses of AGS-003 correlated with survival. Importantly, these CTL consist of proliferating CTL secreting multiple cytokines with lytic activity. We further identified a pre-treatment CTL signature and post-treatment CTL signature, with both subsets lacking the CD28 receptor, as inversely correlated with survival. One patient treated long term with AGS-003 maintained multi-functional CD28+CCR7+CD45RA- CTL outwards to 3 years post-treatment resulting in patient survival in excess of 30 months.

## Conclusion

These observations confirm the in vivo mechanism of action of AGS-003 and provide early biomarkers that could predict long-term clinical outcome. BTSVQ analysis identified multi-functional CTL correlating with clinical outcome. To the best of our knowledge, this is the first report correlating changes in the magnitude of an adaptive immune response post DC immunotherapy with clinical outcome. Data presented herein shows the durability of the AGS-003 induced immune response, present after quarterly dosing with AGS-003. Furthermore, our immune monitoring platform will serve as the basis to correlate immune responses and clinical efficacy in RCC patients receiving AGS-003 in the ongoing randomized Phase 3 ADAPT study using AGS-003 in combination with standard treatment.

